# Difficult strabismus surgery following subconjunctival silicone oil migration: a case report

**DOI:** 10.1097/RC9.0000000000001128

**Published:** 2026-10-13

**Authors:** Majid Farvardin, Mohammad Javad Vahidifard, Hajar Farvardin

**Affiliations:** a Poostchi Ophthalmology Research Center, Department of Ophthalmology, School of Medicine, Shiraz University of Medical Sciences, Shiraz, Iran; b Farvardin Ophthalmology Research Center, Farvardin Eye Clinic, Shiraz, Iran

**Keywords:** complication, conjunctiva, silicone oil, silicone oil migration, strabismus, vitreoretinal surgery

## Abstract

**Introduction and importance:**

Subconjunctival migration of silicone oil is a rare but clinically significant complication following vitreoretinal surgery. The resulting inflammation and fibrosis can increase the difficulty of subsequent ocular operations, such as strabismus surgery. Understanding this phenomenon is crucial for preventing intraoperative complications and ensuring better surgical outcomes.

**Case presentation:**

A 26-year-old female presented with sensory exotropia in the right eye following multiple vitreoretinal surgeries involving silicone oil tamponade. During strabismus surgery, multiple subconjunctival cysts filled with silicone oil and extensive fibrosis were encountered, particularly around the rectus muscle insertions. The procedure required extensive dissection and hemostasis, prolonging operative time. Despite intraoperative challenges, the postoperative ocular alignment achieved orthotropia in the primary position, with satisfactory cosmetic results.

**Clinical discussion:**

Silicone oil is widely used as an internal tamponade in complex retinal detachment surgeries. Migration of silicone oil to extraocular tissues can occur through unsutured sclerotomies, wound dehiscence, or glaucoma drainage devices. Such migration may cause localized granulomatous inflammation, fibrosis, and adhesions of extraocular structures, complicating future surgeries. In this case, repeated vitreoretinal interventions and postoperative ocular hypertension were likely predisposing factors. Awareness of this potential complication is essential to minimize operative risk and optimize outcomes.

**Conclusion:**

Patients with a history of silicone oil tamponade and multiple vitreoretinal procedures are at higher risk of surgical complications during routine strabismus surgery. Surgeons should anticipate difficult tissue handling, prolonged operative time, and potential bleeding. Preventive measures, such as proper sclerotomy closure and management of postoperative hypertonia, are crucial for preventing silicone oil migration.

## Introduction

Strabismus, or ocular misalignment, is a recognized complication following vitreoretinal procedures such as scleral buckling and pars plana vitrectomy^[^1–4^]^. The underlying mechanisms include sensory strabismus, extraocular muscle damage, redirected muscle forces, mass effect of explants, and adherence syndrome^[^1,5,6^]^. Patients with poor vision – predisposing to sensory deviation – and those who undergo multiple surgeries are at particularly high risk^[^5,6^]^.

HIGHLIGHTS

- Silicone oil is frequently used as an internal tamponade during vitrectomy surgeries.
- Silicone oil migration into the subconjunctival space or other neighboring structures may occur after vitreoretinal procedures.
- Silicone oil migration into adjacent structures can complicate routine strabismus surgery.
- Strabismus surgeons should anticipate a challenging surgery when scheduling surgery for patients who have undergone multiple vitreoretinal procedures.

When silicone oil is used as an internal tamponade, it may unintentionally migrate into the subconjunctival space and around the extraocular muscles and contribute to adherence syndrome^[^2^]^. Silicone oil migration has also been reported in the orbit^[^7–9^]^, eyelid^[^10–12^]^, and even the central nervous system^[^13^]^. Such migration provokes localized granulomatous inflammation and fibrosis, which may restrict ocular motility and, critically, substantially complicate any subsequent strabismus correction^[^2,3^]^.

To our knowledge, no previous case report has specifically addressed the intraoperative challenges that strabismus surgeons face when operating on a previously vitrectomized eye with extensive subconjunctival silicone oil migration. This report describes the difficult surgical dissection, unexpected fibrosis at the rectus muscle insertions, and prolonged bleeding encountered during routine recession-resection surgery in such a patient, and offers practical management recommendations as well as preventive strategies.

## Presentation of case

A 26-year-old woman with anisometropic amblyopia and high myopia in the right eye presented to our strabismus referral clinic with sensory exotropia. Over the preceding 5 years, she had undergone four vitreoretinal procedures in the right eye: (1) Lens-sparing deep vitrectomy with silicone oil tamponade and 360-degree barrier laser for rhegmatogenous retinal detachment; (2) Repeat deep vitrectomy, silicone oil exchange, phacoemulsification, and intraocular lens implantation for sub-silicone oil retinal detachment with a macular hole; (3) Silicone oil removal 6 months later; and (4) Silicone oil re-injection for recurrent retinal detachment with a large inferior retinal tear. All episodes of postoperative ocular hypertension were managed medically with topical drops. Her right-eye visual acuity remained hand motions (left eye 20/20). Cycloplegic refraction was −1.00/−3.00 × 170° in the right eye and −3.75/−1.00 × 10° in the left eye. Intraocular pressure was 20 and 17 mmHg in the right and left eyes, respectively. Slit-lamp examination revealed multiple shiny subconjunctival silicone oil cysts in all quadrants of the right eye, and dilated fundus examination showed an attached retina. Ocular motility testing demonstrated a 35-prism-diopter sensory exotropia with mild limitation of both abduction and adduction (−1).

A monocular recess-resect procedure was planned because sensory exotropia of this magnitude typically cannot be fully corrected by maximal unilateral lateral rectus recession alone. The planned dosages were lateral rectus recession of 10 mm and medial rectus resection of 4 mm. The dosage modification was based on standard dose–response estimates for sensory exotropia, in which each millimeter of lateral rectus recession corrects approximately 2 prism diopters and each millimeter of medial rectus resection corrects approximately 3 prism diopters. The senior consultant (Prof. M. F.) decreased the medial rectus resection dosage compared with standard recess-resect tables to avoid induced palpebral fissure narrowing and achieve a better cosmetic outcome. Under general anesthesia, intraoperative forced duction testing was mildly positive for both abduction and adduction. Two large silicone oil-filled cysts were present in the supratemporal and supranasal quadrants; multiple smaller nodules and cysts were found beneath the conjunctiva, within Tenon’s capsule, and at the insertion sites of the medial and lateral rectus muscles (Figs 1 and 2). The cysts were firmly adherent to surrounding tissues, and the underlying sclera appeared thinner than normal, likely related to the patient’s high myopia. The strabismus surgeon avoided unnecessary manipulation of the cysts to minimize the risk of scleral perforation. The procedure was complicated by diffuse oozing and excessive bleeding, which required repeated cauterization, extended the operative time to approximately three times that of a routine recess-resect case, and increased the number of cotton applicators used to five times the usual amount. An adjustable suture technique was deliberately not employed. In an eye with extensive subconjunctival silicone oil cysts, fragile and hypervascular fibrotic tissue planes, and diffuse intraoperative oozing, conjunctival closure and postoperative suture manipulation would have been substantially more difficult and considerably less predictable. Manipulating the conjunctiva in the early postoperative period risked exposing or rupturing the silicone oil cysts, provoking further granulomatous inflammation, and precipitating renewed bleeding.

**Figure 1.**
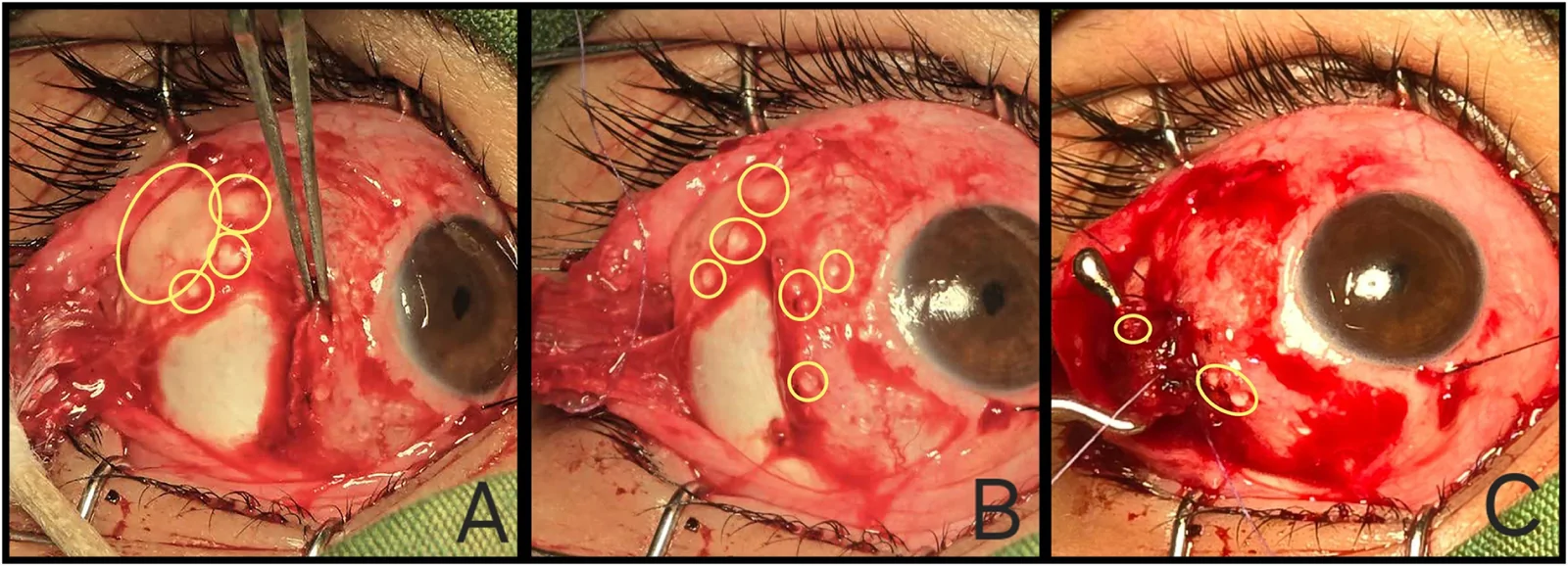
A large silicone oil-filled cyst in the superotemporal quadrant (A), along with silicone oil migration into the subconjunctival space around the lateral rectus muscle (B), causing excessive bleeding (C).

**Figure 2.**
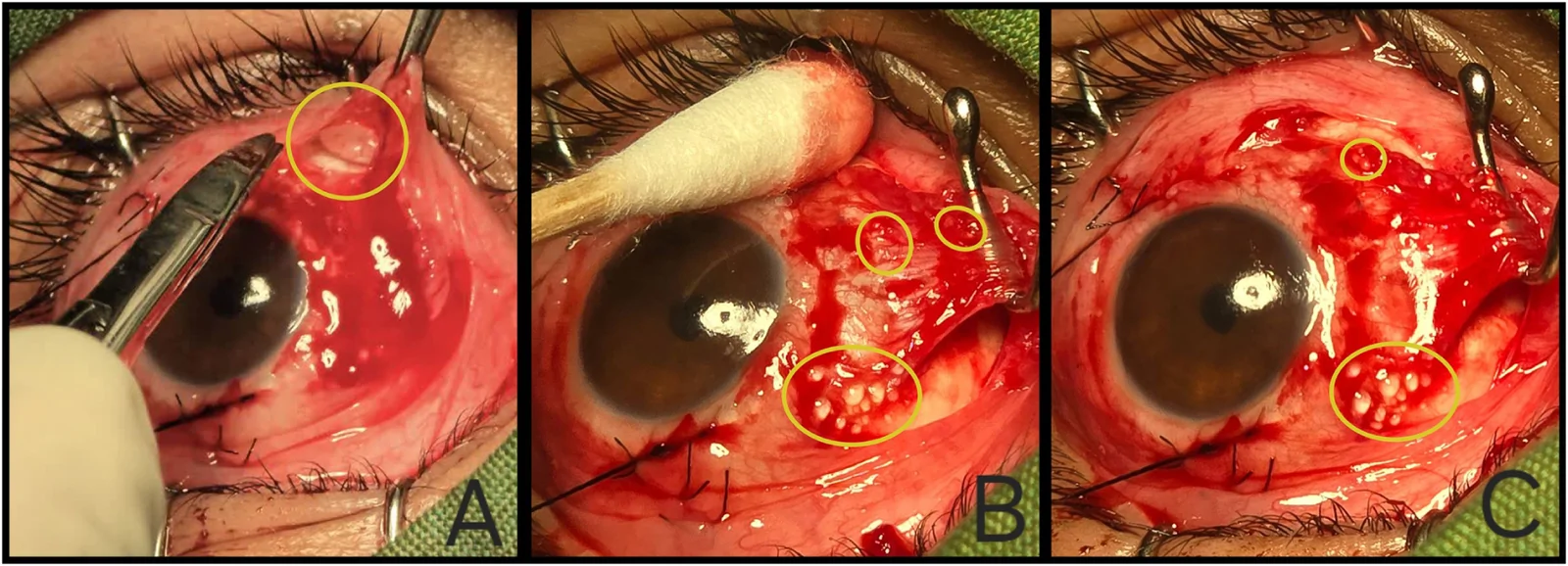
Large silicone oil-filled cyst in the superonasal quadrant (A), with silicone oil migration into the medial rectus muscle (B) and the medial rectus muscle insertion site (C).

Postoperatively, the patient maintained primary-position orthotropia throughout a 6-month follow-up period. No wound leakage, infection, excessive inflammation, new cyst formation, or palpebral fissure asymmetry were observed. Beyond the objective alignment results, the patient reported a meaningful improvement in her psychosocial well-being and increased confidence in social and professional interactions due to the improved cosmetic appearance of her eye.

## Discussion

A variety of tamponade agents are employed in vitreoretinal surgery; silicone oil is reserved for the most complex retinal detachments^[^5^]^. Unintended subconjunctival migration of silicone oil occurs in approximately 2.7% of vitrectomized eyes, predominantly via two routes: unsutured small-gauge sclerotomies – which have become standard practice owing to reduced operative time and enhanced patient comfort – and glaucoma drainage devices^[^3,14–17^]^.

Certain eyes are predisposed to this complication, including those that have undergone multiple vitreoretinal procedures, possess a staphylomatous or intrinsically thin sclera, have suffered postoperative infection or inflammation with scleral melting, or have experienced intraocular pressure spikes that induce wound dehiscence and cheese-wiring of sclerotomy sites^[^2,4,7–18^]^. The present patient exhibited several of these risk factors simultaneously: four vitreoretinal interventions, documented episodes of postoperative ocular hypertension, and an underlying highly myopic, thinner-than-normal sclera. This clustering of risk factors likely explains the unusually extensive subconjunctival and peri-muscular silicone oil dissemination found at the time of strabismus surgery^[^19^]^.

While silicone oil migration is well recognized, previous reports have focused on its incidence and anatomical distribution; very few have provided a detailed, practical account of the intraoperative difficulties it creates for the strabismus surgeon or offered a structured management strategy. The present case is distinguished by its systematic description of the operative challenges and by the formulation of experience-based recommendations. The fibrosis enveloping the rectus muscle insertions, the scleral thinning, and the extensive bleeding – prolonging the operation to three times its usual duration – collectively underscore that such cases cannot be approached as routine recess-resect procedures. Importantly, the cysts were deliberately left intact; their rupture or excision was avoided to minimize the dual risks of scleral perforation and exacerbation of postoperative inflammation and fibrosis. Despite these obstacles, primary-position orthotropia was achieved and remained stable throughout the six-month follow-up period, demonstrating that satisfactory alignment is attainable when the unique difficulties of the scenario are anticipated and respected.

Adjustable sutures are frequently advocated in strabismus surgery when postoperative alignment is uncertain, as they permit titration of the final position in the early postoperative period. We deliberately did not employ an adjustable suture technique in this case. In an eye with extensive subconjunctival silicone oil cysts, fragile and hypervascular fibrotic tissue planes, and diffuse intraoperative oozing, conjunctival closure and postoperative suture manipulation would have been substantially more difficult and considerably less predictable. Manipulating the conjunctiva in the early postoperative period risked exposing or rupturing the silicone oil cysts, provoking further granulomatous inflammation, and precipitating renewed bleeding. Therefore, in such cases, we favor accurate preoperative planning with fixed sutures and a deliberate, slightly conservative dosage strategy rather than relying on postoperative adjustment.

Based on this experience, we suggest the following surgical considerations when scheduling strabismus correction for an eye with clinical evidence of silicone oil migration:

1. Plan for a substantially longer and more difficult procedure. The combination of fibrosis and hypervascularity can triple the usual operative time; the surgical team and anesthetist should be informed accordingly.
2. Anticipate diffuse bleeding. Diffuse oozing from inflamed, fibrotic tissue planes is common and may be refractory to simple pressure. Adequate cauterization and topical hemostatic agents, if available, should be readily accessible.
3. Avoid unnecessary manipulation of silicone oil cysts. The cysts are often firmly adherent to the underlying sclera, which may be abnormally thin. Attempts to excise or decompress them carry a risk of scleral perforation and may provoke additional granulomatous inflammation and fibrosis. Accepting their presence and working around them is safer.
4. Counsel the patient about the elevated risks. The patient should understand that the operation is more challenging than a conventional strabismus procedure and carries a higher likelihood of intraoperative complications and a more uncomfortable postoperative recovery.
5. Consider preoperative orbital imaging. Although imaging was not obtained in this case, it may help map the extent of cysts and assess scleral integrity, allowing for more precise surgical planning. Each modality has distinct advantages and limitations. High-resolution ultrasound biomicroscopy provides excellent detail of the anterior segment, conjunctiva, and extraocular muscle insertions, but it is operator-dependent, requires contact with the ocular surface, may not visualize the full extent of posterior orbital involvement, and may be uncomfortable for some patients. Computed tomography offers broader orbital coverage and is readily available, but it exposes the patient to ionizing radiation. Magnetic resonance imaging avoids radiation and can characterize orbital and intracranial extension, but it is expensive and less accessible. In our patient, the cysts were clinically evident and anterior, and surgery proceeded without imaging; in future cases with suspected deep orbital or posterior involvement, preoperative imaging would be reasonable.

From a preventive standpoint, the two most impactful measures rest with the vitreoretinal surgeon. First, meticulous closure of sclerotomy sites should be performed at the end of every vitreoretinal procedure; even with small-gauge instrumentation, this can substantially reduce the exit of silicone oil – particularly in re-operations where scleral elasticity is compromised. We recommend suturing sclerotomies with absorbable sutures in these high-risk situations rather than relying on unsutured self-sealing wounds. Second, postoperative intraocular pressure must be monitored closely and controlled aggressively. Medical management with topical aqueous suppressants and, where appropriate, oral carbonic anhydrase inhibitors is the first line; however, if intraocular pressure remains uncontrolled, silicone oil decompression, glaucoma drainage device implantation, and early referral to the glaucoma service should be considered without delay. In the present case, unsutured sclerotomies and documented episodes of postoperative ocular hypertension coexisted, making extensive silicone oil migration entirely plausible. We therefore recommend that vitreoretinal surgeons maintain a low threshold for involving the glaucoma service whenever postoperative intraocular pressure rises, so that pressure spikes are treated before they can promote wound dehiscence and oil egress.

This report has limitations, including the absence of orbital imaging and histopathological confirmation, the absence of a formal quality-of-life instrument, and the relatively short six-month follow-up. However, the objective of this case is not to provide long-term epidemiological data but to vividly illustrate the intraoperative reality of operating in a silicone-oil-infiltrated field and to equip strabismus surgeons with practical strategies for managing it. With meticulous planning, respect for the altered tissue planes, and adherence to the principles outlined above, safe and effective ocular alignment can be achieved.

## Conclusion

This case demonstrates that strabismus surgery in an eye with extensive subconjunctival silicone oil migration is distinctly challenging. Nevertheless, by anticipating a substantially longer operative time, avoiding unnecessary manipulation of the silicone oil cysts, and employing meticulous hemostasis, satisfactory primary-position orthotropia can be achieved and maintained. Preventive measures such as meticulous sclerotomy closure and aggressive control of postoperative intraocular pressure remain the most effective strategies to reduce the risk of silicone oil migration. When these precautions are combined with careful preoperative planning, safe and effective ocular realignment is attainable.

## Data Availability

All data generated during this study are included in the published article.

## Research registration unique identifying number (UIN)

32263.

## SCARE statement

This case report has been reported in line with the SCARE 2025 criteria. The Revised Surgical Case Report (SCARE) guideline: An update for the age of Artificial Intelligence was developed by Kerwan A, Al-Jabir A, Mathew G, Sohrabi C, Rashid R, Franchi T, Nicola M, Agha M, Agha RA and published in *Premier Journal of Science*. 2025;10:100079.
